# Genome-wide CRISPR knockout screen identifies ZNF304 as a silencer of HIV transcription that promotes viral latency

**DOI:** 10.1371/journal.ppat.1008834

**Published:** 2020-09-21

**Authors:** Simona Krasnopolsky, Alona Kuzmina, Ran Taube

**Affiliations:** The Shraga Segal Department of Microbiology Immunology and Genetics, Faculty of Health Sciences, Ben-Gurion University of the Negev, Beer-Sheva, Israel; Imperial College London, UNITED KINGDOM

## Abstract

Despite the widespread use of anti-retroviral therapy, human immunodeficiency virus (HIV) still persists in an infected cell reservoir that harbors transcriptionally silent yet replication-competent proviruses. While significant progress has been made in understanding how the HIV reservoir is established, transcription repression mechanisms that are enforced on the integrated viral promoter have not been fully revealed. In this study, we performed a whole-genome CRISPR knockout screen in HIV infected T cells to identify host genes that potentially promote HIV latency. Of several top candidates, the KRAB-containing zinc finger protein, ZNF304, was identified as the top hit. ZNF304 silences HIV gene transcription through associating with TRIM28 and recruiting to the viral promoter heterochromatin-inducing methyltransferases, including the polycomb repression complex (PRC) and SETB1. Depletion of ZNF304 expression reduced levels of H3K9me3, H3K27me3 and H2AK119ub repressive histone marks on the HIV promoter as well as SETB1 and TRIM28, ultimately enhancing HIV gene transcription. Significantly, ZNF304 also promoted HIV latency, as its depletion delayed the entry of HIV infected cells into latency. In primary CD4^+^ cells, ectopic expression of ZNF304 silenced viral transcription. We conclude that by associating with TRIM28 and recruiting host transcriptional repressive complexes, SETB1 and PRC, to the HIV promoter, ZNF304 silences HIV gene transcription and promotes viral latency.

## Author summary

Antiretroviral therapy has significantly decreased the morbidity and mortality associated with HIV infection. However, a complete cure remains out of reach, as HIV persists in a cell reservoir that is highly stable in the face of therapy. While developing novel therapeutic strategies to eliminate the reservoir is a well-recognized goal, knowledge of the molecular events that establish HIV latency is still not complete. To obtain insights into the silencing mechanisms of HIV gene transcription and the establishment of viral latency, a genome-wide CRISPR screen was employed to identify host factors that control viral latency. We identified zinc-finger protein 304 (ZNF304) and showed that through association with TRIM28, it recruits the histone methyltransferases SETB1 and PRC to deposit repressive marks on chromatin of the HIV promoter, thereby facilitating the silencing of viral gene transcription. Moreover, we found that depletion of ZNF304 expression activated HIV gene expression, while ZNF304 overexpression repressed viral gene transcription both in a T cell line and in primary CD4^+^ cells. Finally, our study showed that ZNF304 is also involved in modulating HIV latency, as its depletion delayed entry of the virus into a latency state. Our results offer an additional mechanistic explanation for how host histone repression complexes are tethered to the HIV promoter to promote chromatin compaction, thereby defining a potentially new target for perturbing the establishment of the viral reservoir.

## Introduction

The introduction of antiretroviral therapy (ART) has limited the spread of the human immunodeficiency virus (HIV) and has significantly improved the clinical outcomes associated with this viral infection. Yet, a complete cure for HIV infection remains out of reach, as the transcriptionally silent but replication-competent provirus persists in a long-lived cell reservoir, comprised mainly of memory CD4^+^ T cells. This reservoir is highly stable and is refractory to antiviral drugs and to the effect of the immune response, and thus constitutes a major obstacle towards complete eradication of HIV infection [1–11]. Moreover, recent data show that there is a clonal homeostatic expansion of infected memory T cells, suggesting that the latent state is dynamic, with low levels of HIV reactivation and cell death [12, 13]. As elimination of the HIV reservoir remains a challenge, there is an urgent need for a better understanding of the molecular events that establish viral latency. Among the pathways that control the establishment of the HIV reservoir, epigenetic constraints and mechanisms that suppress proviral gene transcription are prominent [14–18]. One approach that has been suggested for eliminating the HIV reservoir is the “shock-and-kill” strategy, which employs latency-reversing agents (LRAs) to specifically activate resting infected cells, exposing them to the immune system and to the effects of ART [19–23]. This approach, which is based on the successful use of LRAs as modulators of chromatin architecture, has, however, regretfully failed to display clinical efficacy in vivo, thereby prompting the search for alternative therapies [16, 24–26]. Nonetheless, despite considerable progress, new and improved strategies for eradicating the HIV reservoir remain out of reach, and the pathways governing proviral silencing are yet to be fully understood [1, 27].

Zinc finger proteins (ZNFs) are one of the most diverse groups of eukaryotic DNA binding proteins, constituting the single largest class of transcription factors encoded in the human genome [28]. They display a broad pattern of expression and have been linked to diverse biological processes, such as genomic imprinting, RNA metabolism, cell development and differentiation, metabolic control, and meiotic recombination [29–34]. ZNFs include two conserved motifs, an N-terminal Kruppel-associated box (KRAB) domain that recruits protein partners that can modulate chromatin architecture and gene expression, and a C-terminal C2H2 zinc finger region that mediates binding of ZNF to DNA. The KRAB domain associates with TRIM28/KAP1 (tripartite motif-containing 28/KRAB-associated protein 1), in which the tripartite-motif (TRIM) serves as a scaffold for recruiting heterochromatin-inducing lysine histone methyltransferases (KHMTs), such as SETB1, heterochromatin protein 1 (HP1), Suv39H1, G9a and a G9a-like protein (GLP) [35–37]. Accordingly, ZNF809-TRIM28 interactions have been reported to initiate epigenetic silencing during early development and genomic reprogramming in transposable elements, endogenous retroviruses and embryonic stem cells through recruiting SETB1 and HP1 KHMTs [37, 38, 39, 40]. Similarly, the Yin Yang 1 (YY1) ZNF binds the long terminal repeats (LTR) of retroviruses and recruits TRIM28 [41] and additional key silencing proteins, such as HP1 [39, 42], EBP1 [43], NuRD and the H3K9me3-ESET [44–47]. In the specific context of HIV, ZNF10 has previously been reported to repress viral gene transcription through recruiting TRIM28, SETDB1 and HP1 [48].

Here, we report a whole-genome CRISPR knockout (KO) screen in HIV-infected T cell models with the aim of identifying host factors that control viral gene silencing and viral latency. Our screen identified several candidate partners that are potentially involved in promoting HIV latency. Among them, ZNF304 was shown to occupy the HIV promoter and to promote viral gene transcription repression in T-cell models and in primary CD4^+^ T cells. Initially, ZNF304 was identified as a regulator of lymphoid cell activation [49], but it was later reported to promote multiple oncogenic pathways and to play an important role in cell survival, migration and invasion. In human ovarian cancer, ZNF304 has been linked to increased tumor metastasis and overall poor survival by acting as a transcriptional regulator of β1 integrin and ultimately preventing anoikis [50]. In colorectal cancers, ZNF304 is known to silence the *INK4-ARF* promoter, establishing a CpG island phenotype and recruiting a co-repressor complex that includes the DNA methyltransferase DNMT1 [51]. However, to date, the involvement of ZNF304 in controlling HIV gene silencing has not been suggested.

Our work joins several other CRISPR screens that aimed to identify cell partners for HIV [52–55]. Mechanistically, we found that ZNF304 associates with TRIM28 and potentially recruits heterochromatin-inducing complexes, such as polycomb repressing complex (PRC1/2) and SETB1 histone lysine methyltransferases, to the HIV promoter, thereby depositing H3K27me3, H2AK119Ub and H3K9me3, respectively. Importantly, we showed that ZNF304 promotes HIV latency, as its depletion delayed re-entry of HIV into a latency state. We therefore concluded that ZNF304 acts a modulator of HIV gene expression and viral latency and promotes HIV transcription repression and viral latency through the recruitment of host transcription repressor complexes to the HIV promoter.

## Results

### CRISPR knock-out screen identifies host factors that activate latent HIV

**Fig 1:**
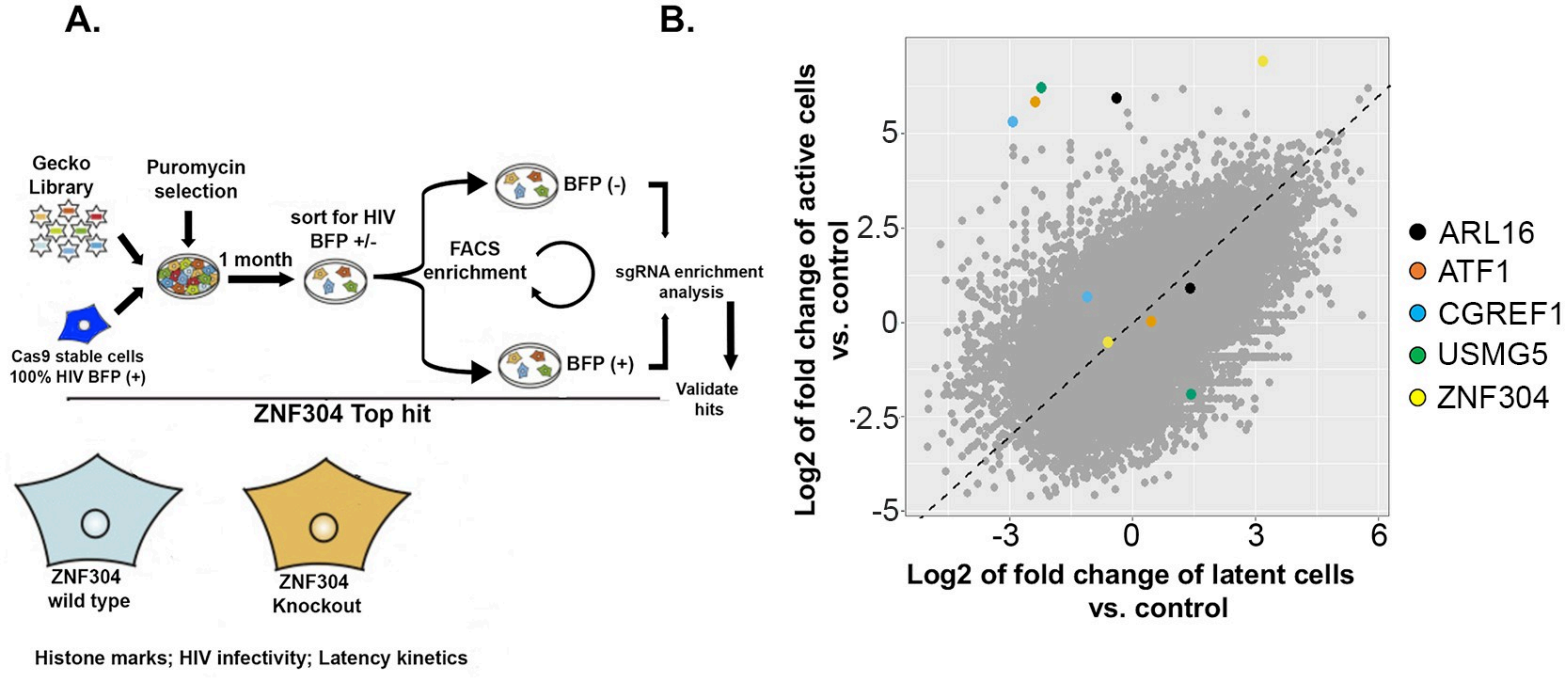
Genome-wide CRISPR knockout screen for identifying host factors that promote HIV latency. **A**. Schematic representation of the genome CRISPR KO screen towards identifying host factors that modulate HIV gene transcription and viral latency by using the GeCKO library. **B**. Scatter plot showing enrichment of sgRNA in activated or latent cells relative to control unsorted latent cells. Plot was generated by the CRISPR analyzer. Differences in enrichment were calculated as log2-normalized fold of change. The indicated colored sgRNAs were significantly enriched in our screen; of these the highest-ranking gene was ZNF304. This gene was detected across two independent screens and was thus chosen for further validation and mechanistic investigation. Dots near the diagonal represent sgRNA with no enrichment between latent or activate state of HIV.

To systematically identify human host factors that support the establishment of HIV latency, we performed a whole-genome CRISPR KO screen in an HIV-infected Jurkat T cell line (Fig 1). Cas9 stable Jurkat cells were initially generated by transduction of a lentivirus expressing Cas9, followed by selection with blastocydin (S1 Fig). Cas9-stable cells were then transduced with a pseudotyped HIV-blue fluorescent protein (BFP) lentivirus, where BFP expression correlated with HIV-1 gene expression. Two days later, cells were sorted on the basis of their BFP expression to obtain a pure population of BFP-expressing cells. Thereafter, cells were transduced, at a low multiplicity of infection (MOI) of 0.3, with the Genome-scale CRISPR Knock-Out (GeCKO) whole genome library (details on the GeCKO library and the screen protocol are given in Materials and Methods) [56], and then subjected to puromycin selection to ensure that all surviving cells stably expressed the GeCKO sgRNA library. Thereafter, cells were cultured for up to one month, allowing them to enter latency, as determined by a decrease in BFP expression. Cells were then FACS-sorted and separated into two groups as follows: (i) those that did not express BFP and thus silenced HIV gene expression, designated BFP(–), which constituted 45% of the entire live population, and (ii) those that expressed a transcriptionally active provirus and could not enter latency, designated BFP(+), which constituted 44% of the entire live population. The control cells for this screen were Cas9-Jurkat-HIV-BFP cells that also harbored the GeCKO library, but were harvested immediately after completion of puromycin selection and were thus not sorted. In this group, close to 90% of the cells expressed HIV-BFP. For the experimental group and for the unsorted control group of cells, 12 million cells (×100 the size of the GeCKO library) of each were collected, and genomic DNA was extracted for PCR amplification of the region that flanked the single guide (sg)RNA. Amplicons were purified and directly sequenced by next-generation sequencing (NGS). The CRISPR analyzer algorithm was used to analyze the amplicon sequences and to identify enriched sgRNAs in experimental versus control unsorted cells. Our screening protocol (illustrated in Fig 1A) found that the sgRNA distribution differed between the HIV infected cells and the control unsorted cells, with a subset of sgRNAs that were enriched in the experimental group relative to the control. The sgRNAs that were found to be significantly enriched were obtained in two independent screens and sequences, and sgRNAs of the top hits are indicated in different colors on Fig 1B. Among the top candidates, the highest-ranking gene was ZNF304.

### Functional validation of the CRISPR knock-out screen

**Fig 2:**
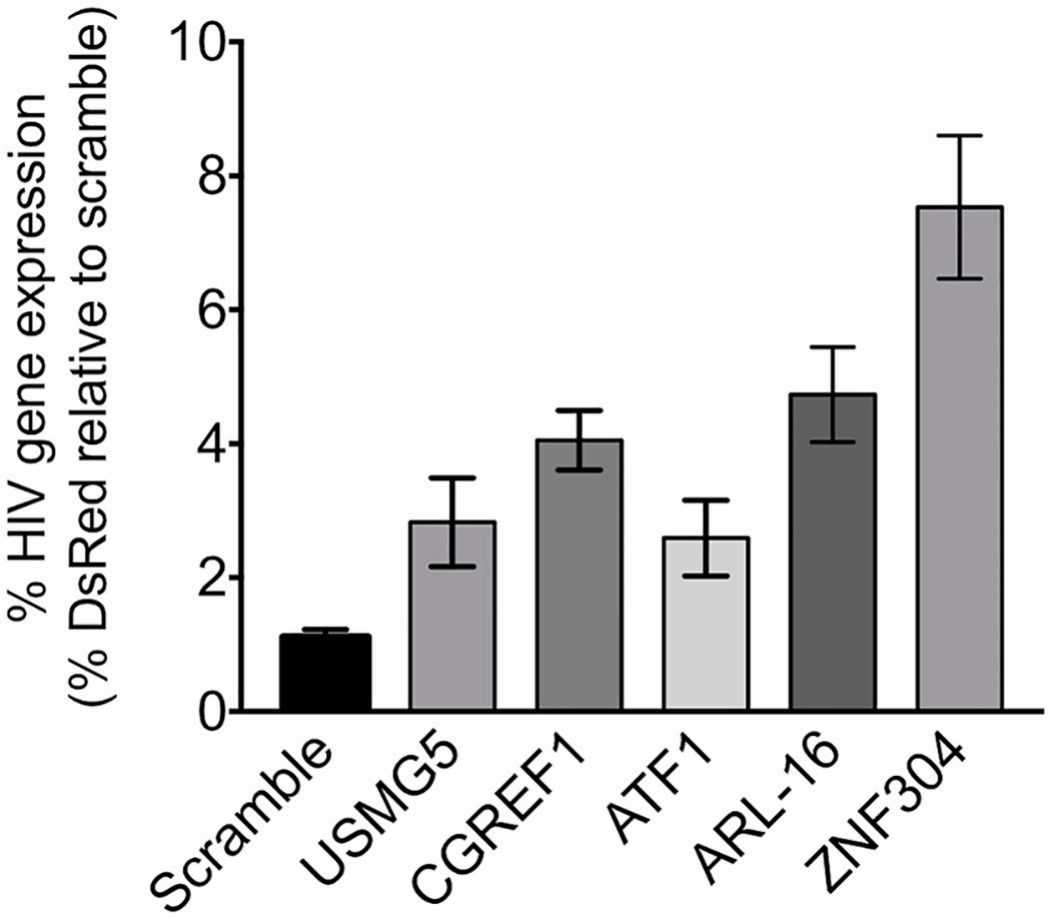
Functional validation of the HIV CRISPR knockout screen. Jurkat T cells were transduced with a lentivirus that drives the expression of Cas9 and sgRNAs that target the indicated gene. Following selection, polyclonal cells were transduced with HIV-DsRed, and 30 days post transduction cells were subjected to FACS analysis. DsRed expression in transduced cells was determined and is presented relative to cells expressing scramble sgRNA set to 1. Error bars represent means ± SD of three independent reactions.

To validate the functional significance of the top gene candidates that were identified in our screen, their expression was separately depleted in Jurkat T cells. To this end, Jurkat-Cas9 stable cells were transduced with sgRNAs, each specifically targeting one of the top candidates, namely, ARL-16, ATF1, CGREF1, USMG5, and ZNF304. For each gene, several different sgRNAs were tested to confirm optimal KO of gene expression. As a control for gene KO specificity, cells were also transduced with a scramble sgRNA that did not target any specific host gene. Cells were then selected with puromycin, until control cells that did not express the sgRNA cassette died. Polyclonal cells that survived the drug selection were transduced with HIV-DsRed lentivirus, and viral gene expression was monitored 30 days post transduction by FACS. Viral gene expression was calculated relative to DsRed expression in cells that harbored control scramble sgRNA (Fig 2). Our results showed that depletion of each of the candidates led to HIV gene activation, which was two- to six-fold higher than that of the control cells expressing a scramble sgRNA. Of these candidates, depletion of ZNF304 expression had the most marked effect on HIV gene expression, namely, six-fold relative to the transcription levels shown in cells that expressed scramble sgRNA, and we thus focused our study on this protein.

### ZNF304 expression levels and promoter occupancy are elevated upon cell activation and correlate with HIV gene activation

**Fig 3:**
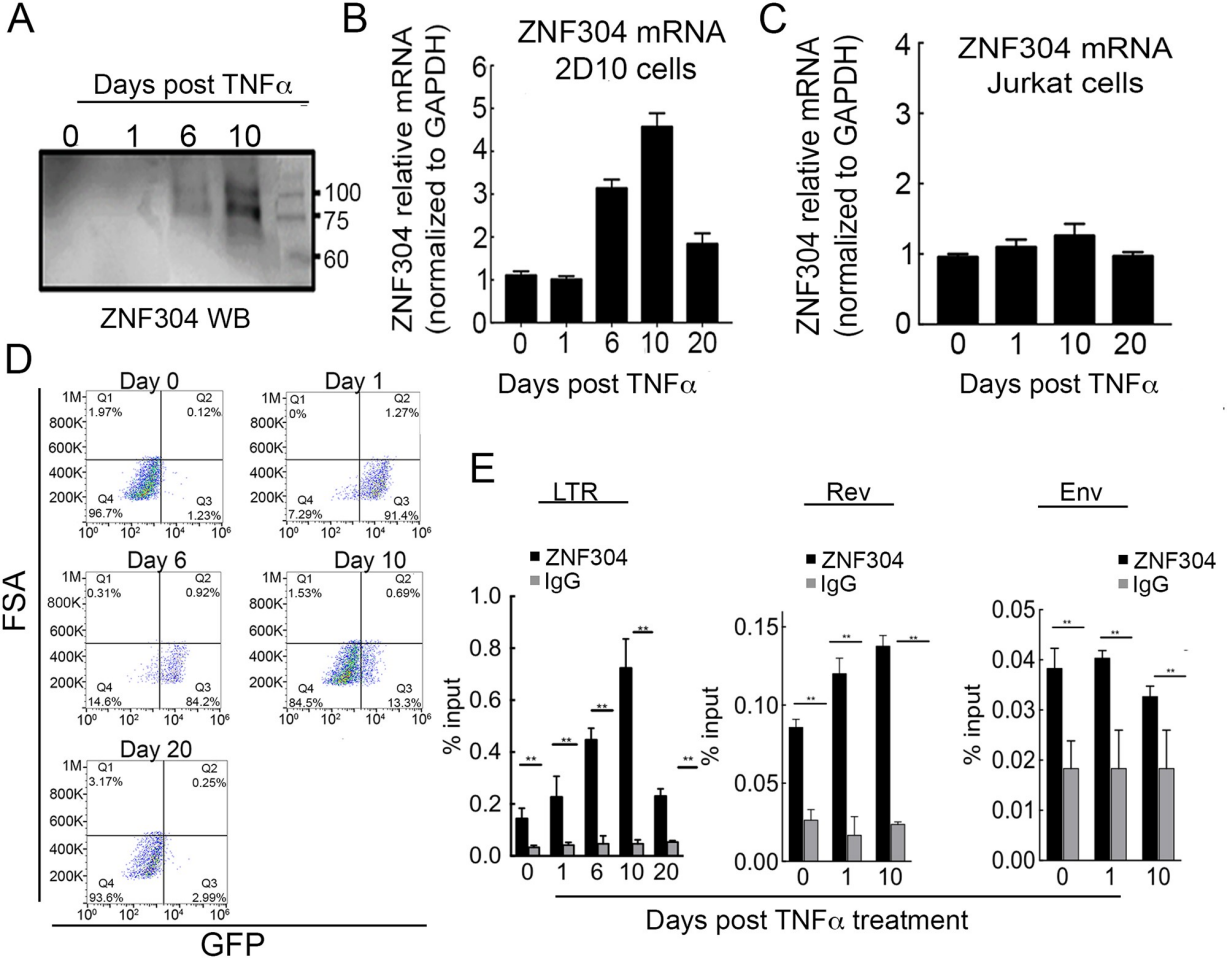
ZNF304 expression levels and promoter occupancy are elevated upon cell activation. **A. ZNF304 protein expression levels following TNFα treatment**– 2D10 cells were treated with TNFα to activate proviral HIV-GFP expression and then subjected to western blot at the indicated times post treatment to monitor ZNF304 expression using ZNF304 IgG. **B. ZNF304 mRNA levels following TNFα treatment**–RNA was extracted from 2D10 cells at the indicated time points post TNFα treatment and then subjected to RT-qPCR to determine ZNF304 mRNA levels relative to GAPDH mRNA. Bar graphs show mean values ± SD of three independent experiments. Asterisks indicate different levels of statistical significance as calculated by a two-tailed Student’s t test (** p≤0.01). **C. ZNF304 mRNA expression levels following cell activation in non-infected cells**–Jurkat cells were treated with TNFα and mRNA levels of ZNF304 were determined at the indicated time points by RT-qPCR. Bar graphs show mean values ± SD of three independent experiments. **D. HIV gene expression following TNFα activation**–FACS dot blot of 2D10 cells showing HIV-GFP expression at the indicated time points following TNFα treatment. **E. ZNF304 occupancy on the HIV genome**–TNFα-treated 2D10 cells were subjected to ChIP-qPCR at the indicated time points post cell activation using ZNF304 IgG. qPCR was performed using primers located on the HIV genome (primer positions are shown in S1 Fig). Bar graphs show mean values ± SD of three independent experiments. Asterisks indicate different levels of statistical significance as calculated by a two-tailed Student’s t test (** p≤0.01).

We followed protein expression and mRNA levels of ZNF304 following activation of latently HIV-infected jurkat T cell line, 2D10, with TNFα. 2D10 harbor an integrated HIV, where *Nef* is replaced by 2deGFP reporter and serve as a model for HIV latency (gene scheme of 2D10 cells is presented in S2A Fig; Fig 3) [57]. Treatment of 2D10 cells with TNFα results in activation of HIV gene expression as monitored by increase of GFP expression. ZNF304 protein expression levels were determined by western blotting, and ZNF304 mRNA levels were monitored by RT-qPCR in latent 2D10 cells, or in 2D10 cells that were treated with 12.5ng/ml TNFα, which activated HIV gene expression (Fig 3A and 3B; S2B Fig). Our results demonstrated that in a latent state of HIV, prior to treatment with TNFα (day 0), ZNF304 mRNA levels were relatively low, and protein levels were undetectable. Upon cell activation with TNFα, ZNF304 mRNA levels increased above background levels, starting at day 6 post activation, and reaching their highest value on day 10 (four times higher than background levels), before returning to background levels on day 20 post TNFα treatment. ZNF304 protein expression followed a similar pattern as that of the mRNA levels, reaching the highest level on day 10 post TNFα treatment, and returning to undetectable levels in the latency state (day 0 and day 20) (Fig 3A and 3B). Interestingly, dot blot FACS analysis of 2D10 cells demonstrated that HIV gene expression, as monitored by GFP expression, preceded ZNF304 expression, reaching 90% GFP(+) at 24 h post TNFα treatment and gradually entering transcription repression starting on day 10 (Fig 3D). To rule out the possibility that TNFα alone can induce ZNF304 expression, we treated Jurkat T cells with TNFα and analyzed mRNA levels of ZNF304. Our results showed that in the absence of proviral HIV, cell activation by TNFα had no effects on ZNF304 mRNA levels, while ZNF304 protein expression levels were undetected (Fig 3C).

We also followed the occupancy of ZNF304 on the HIV promoter following TNFα treatment by using chromatin immunoprecipitation (ChIP)-qPCR (Fig 3E). We focused our analysis on the HIV LTR promoter and on the downstream HIV sequences, *Rev* and *Env* (see primer location–S2A Fig). In addition, the binding sequences of ZNF304 on the HIV genome were predicted based on a support vector machine (SVM) algorithm [50, 58]. Our analysis identified 10 potential ZNF304-binding sites specifically on the HIV LTR promoter, with scores ranging from 25.8 to 13.2, allowing us to focus our analysis on this region (S3 Fig). Our RT-qPCR data showed that following cell activation with TNFα, ZNF304 promoter occupancy reached peak levels on day 10 post TNFα treatment, and returned to background levels (observed on day 0) in the latent state (day 20) (Fig 3E). Furthermore, our, ChIP-qPCR experiments demonstrated that the occupancies of ZNF304 on HIV downstream genes, *Rev* and *Env*, were low relative to the occupancy of ZNF304 on the viral promoter (Fig 3E).

### ZNF304-mediated HIV gene transcription silencing

**Fig 4:**
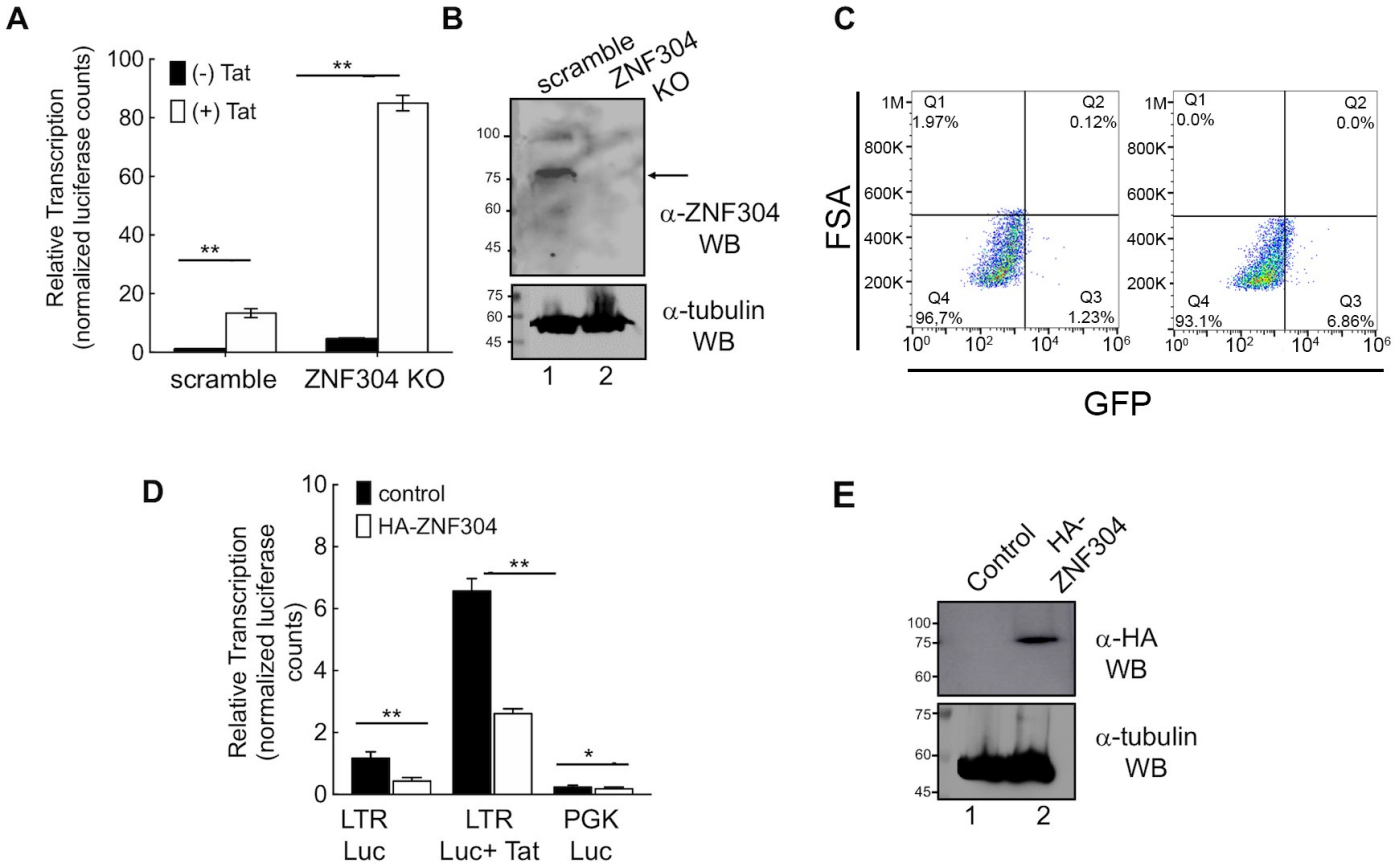
ZNF304 silences HIV gene transcription and modulates viral latency. **A. ZNF304 affects HIV gene transcription**–Jurkat cells in which ZNF304 expression was depleted (ZNF304 KO) or control cells that express scramble sgRNA were transduced with HIV-luciferase (LTR-Luc; black bars). For expressing HIV Tat, cells were further transduced with an HIV-Tat-expressing lentivirus (HIV-Luc+Tat; white bars). Luciferase activity was monitored in control and ZNF304 KO cells forty-eight hours post transduction according to the manufacturer’s protocol. Data are presented relative to luciferase readings (normalized to protein levels) in control cells that express scramble sgRNA set to 1. Bar graphs show mean values ± SD of three independent experiments. Asterisks indicate different levels of statistical significance as calculated by a two-tailed Student’s t test (** p≤0.01). **B**. Western blot analysis for depletion of ZNF304 –Lane 1, control cells expressing scramble sgRNA; Lane 2, ZNF304 KO cells. Protein levels were monitored using ZNF304 IgG. The lower gel represents tubulin western blot, confirming equal protein loading. **C. Involvement of ZNF304 in promoting HIV latency**– 2D10 T cells were depleted of ZNF304 expression by transducing them with a lentivirus driving the expression of a specific sgRNA that targets ZNF304 and Cas9. GFP expression was monitored by FACS. Data is presented as dot blot showing GFP expression in control and ZNF304 KO cells. **D. Overexpression of ZNF304 silences HIV gene transcription**–Control (black bars) or Jurkat cells that stably express HA-ZNF304 (white bars) were transduced with a lentivirus that drives the expression of HIV-LTR-Luc HIV, HIV-LTR-Tat-Luc or PGK Luc. Luciferase readings were determined according to the manufacturer’s protocol and normalized to protein concentrations determined by the Bradford method. Western blot analysis confirmed HA-ZNF304 expression using HA IgG. Asterisks indicate different levels of statistical significance as calculated by a two-tailed Student’s t test (*** p≤0.001; * p≤0.1). **E**. Western blot analysis confirming HA-ZNF304 expression—Lane 1 control cells; Lane 2 cells expressing HA-ZNF304. Western blot was performed with anti-HA IgG. The lower gel represents tubulin expression levels, confirming equal protein loading.

To monitor the effects of ZNF304 on basal and Tat-dependent HIV transcription, we generated Jurkat T cells in which ZNF304 expression was depleted by using CRISPR (S4 Fig confirms the KO sequence of ZNF304). Western blot analysis for ZNF304 expression confirmed protein depletion in the Jurkat cells (Fig 4B). Cells were then transduced with either HIV-LTR-luciferase (Luc) or HIV-Luc+Tat (provided by a second transduction with lentivirus coding for Tat), and viral gene transcription was monitored by determining the luciferase readings relative to control cells that expressed scramble sgRNA (Fig 4A). Our results showed that KO of ZNF304 expression led to an overall activation of HIV gene transcription, which was either Tat independent (black bars in Fig 4A; five-fold activation relative to control cells that expressed scramble sgRNA), or Tat dependent (white bars in Fig 4A; eight-fold relative to control). As expected, in control cells that expressed scramble sgRNA, expression of Tat led to ~10-fold activation of HIV gene transcription relative to Tat-independent basal HIV transcription. Similar activation of Tat was shown in ZNF KO cells. We concluded that depletion of ZNF304 enhanced HIV gene expression (Fig 4A). We also sought to verify the effects of ZNF304 on reactivating HIV latent cells. To this end, we knocked-out ZNF304 expression in 2D10 cells by using CRISPR (Fig 4C). Our FACS dot blot analysis showed that depletion of ZNF304 expression led to ~10-fold activation of HIV, relative to control cells that were transduced with a lentivirus expressing scramble sgRNA (Fig 4C). In addition, we overexpressed ZNF304 in Jurkat cells and monitored effects on HIV gene transcription. Upon ectopic expression of ZNF304, HIV gene expression was silenced, both in the presence and the absence of HIV Tat (Fig 4D). Moreover, ZNF304-mediated silencing of the HIV LTR promoter was specific, as a PGK promoter was not repressed upon expression of ZNF304. Western blot analysis confirmed ZNF304 overexpression in stable cells transduced with HIV-LTR-Luc +Tat (lane 2) relative to control cells (lane 1) (Fig 4E). Similar silencing effects of overexpressed ZNF304 on HIV gene transcription were also shown in other cells lines, i.e., HEK293T and TZM-HeLa cells (S5A, S5B and S5C Fig). Finally, to determine the functional significance of the zinc finger DNA binding motif of ZNF304, we over expressed ZNF304-delta ZNF in Jurkat cells. Cells were then transduced with HIV-Luc and luciferase readings were determined (S5D Fig). To determine the effects of ZNF304-delta ZNF, on Tat transactivation, cells were also transduced with HIV-Tat. Our results demonstrate that ZNF304-delta ZNF had no silencing effects on HIV transcription. This was the case both on basal viral gene transcription and on Tat transactivation. We conclude that the ZNF region of ZNF304 is essential for silencing effects of ZNF304 (S5D Fig).

### ZNF304 recruits the host repressive complex to silence HIV gene transcription

**Fig 5:**
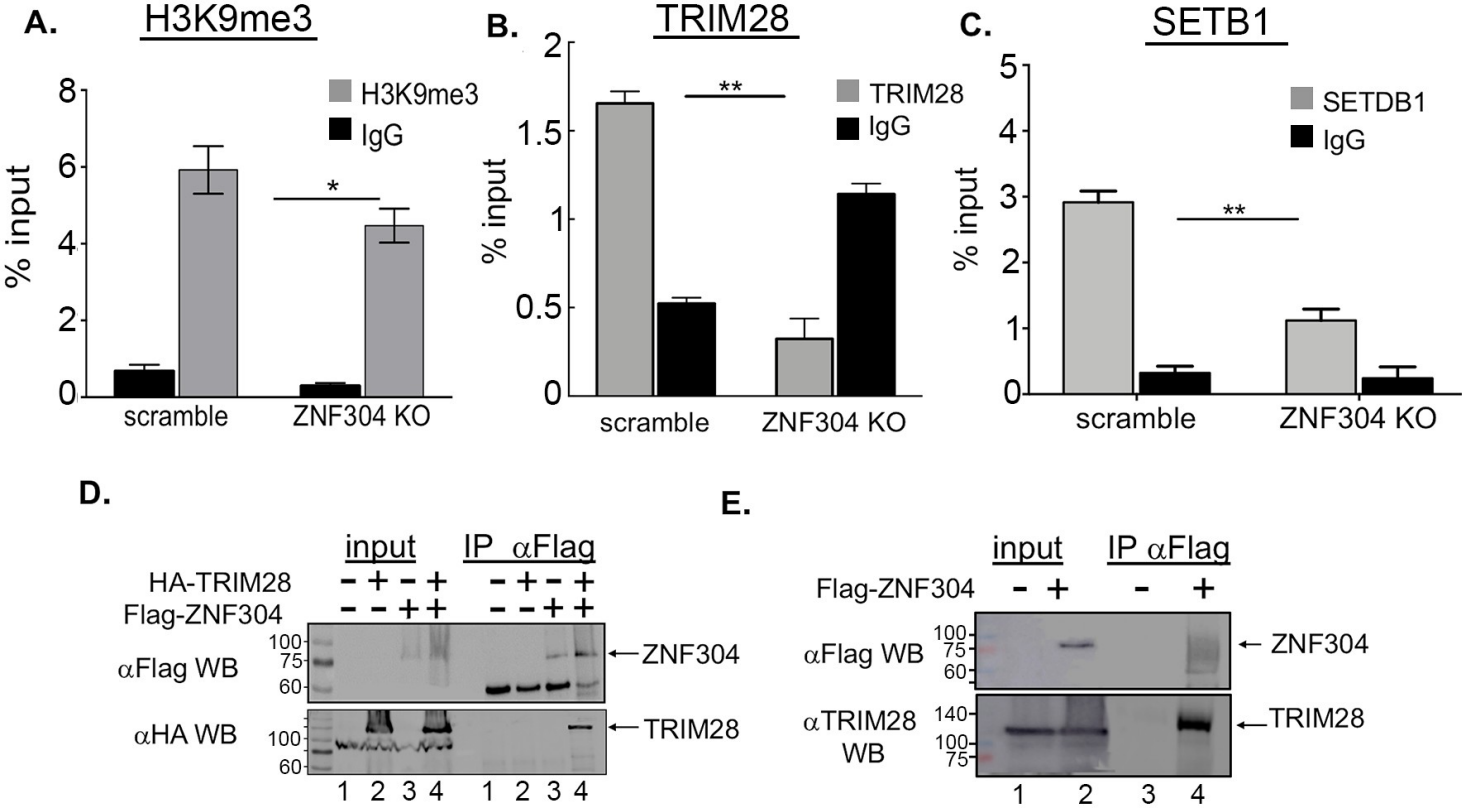
ZNF304 mediates H3K9me3 histone marks on the HIV promoter through TRIM28 and SETB1 to silence HIV gene transcription A-C. ChIP material was isolated from control or ZNF304-depleted T cells—Immuno-precipitation was conducted with H3K9me3 (**A**), TRIM28 (**B**) and SETB1 (**C**). Non-specific rabbit IgG (black bars) was used as control. qPCR on IP samples was conducted with primers located on the HIV promoter, and signals are presented as a percentage of input. Error bars represent means ± SD of three independent qPCR reactions. Asterisks indicate different levels of statistical significance as calculated by a two-tailed Student’s t test (** p≤0.01; * p≤0.1). **D**. **ZNF304 associates with TRIM28 in cells**–HEK293T cells expressing HA-TRIM28 and Flag-ZNF304 were subjected to immunoprecipitation with Flag IgG. IP samples were then subjected to SDS-PAGE and western blot with HA IgG. Input samples represent 10% of the total cell lysate. **E. ZNF associates with endogenous TRIM28 in cells-** HEK293T cells expressing Flag-ZNF304 were subjected to immunoprecipitation with Flag IgG. IP samples were then subjected to SDS-PAGE and western blot with TRIM28 IgG. Input samples represent 10% of the total cell lysate.

We next sought to elucidate the mechanisms by which ZNF304 promotes silencing of HIV gene transcription. We thus monitored the enrichment of common histone heterochromatin marks on the integrated HIV promoter in control or ZNF304-depleted 2D10 cells by ChiP-qPCR (Fig 5). Our results confirmed that ZNF304 depletion resulted in a moderate reduced enrichment of the H3K9me3 histone repressive mark, relative to control cells that expressed scramble sgRNA (Fig 5A). Since TRIM28 recruits SETB1 to the HIV promoter and is known to mediate H3K9me3 through association with ZNFs, we also followed, the occupancy of TRIM28 and SETB1 on the HIV promoter by ChIP-qPCR (Fig 5B and 5C) [59]. Our results indicated that depletion of ZNF304 led to a decreased in occupancy levels of TRIM28 and SETB1 on the HIV promoter, relative to control cells (Fig 5B and 5C). In addition, since it is known that TRIM28 associates with ZNF proteins in human cells and recruits SETB1 to retroviral gene promoters, thereby silencing retroviral gene expression [37, 40, 45, 60], we examined the association between ZNF304 and TRIM28 in HEK293T cells by western blotting (Fig 5D) [61]. Our results verified that ectopically expressed HA-TRIM28 and Flag-ZNF304 associated in cells (Fig 5D). Moreover, interactions between Flag-ZNF304 and endogenous TRIM28 were also demonstrated by western blot using endogenous TRIM28 IgG (Fig 5E). Despite several attempts, we could not detect an interaction between endogenous ZNF304 and TRIM28 proteins, presumably due to the low efficiency of the ZNF304 antibody.

**Fig 6:**
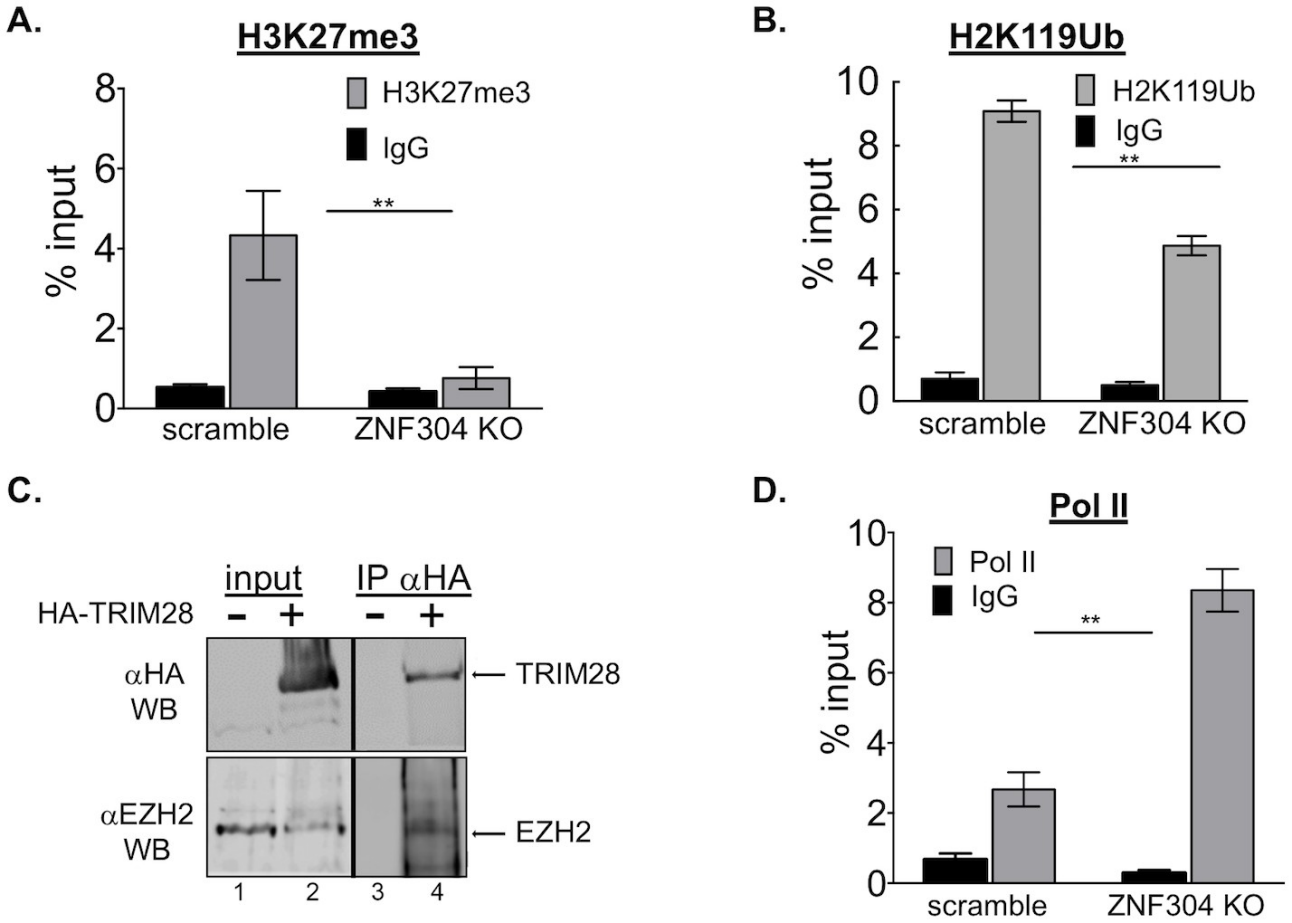
ZNF304 mediates H3K27me3 and H2AK119Ub histone marks on the HIV promoter through TRIM28 and PRC1/2 to silence HIV gene transcription A, B and D. ChIP material was isolated from control or ZNF304-depleted cells. Immuno-precipitation was conducted with H3K27me3 (**A**), H2AK119ub (**B**), or Pol II (**D**). In all experiments, non-specific rabbit IgG was used as the control. qPCR on IP samples was conducted with primers located on the HIV promoter, and signals are presented as a percentage of input. Error bars represent means ± SD of three independent qPCR reactions. Asterisks indicate different levels of statistical significance as calculated by a two-tailed Student’s t test (** p≤0.01). **C**. **TRIM28 associates with EZH2 in cells**–HEK293T cells expressing HA-TRIM28 were subjected to immunoprecipitation with HA IgG or control IgG. IP samples were then subjected to SDS-PAGE and western blot with HA IgG. Input samples represent 5% of the total cell lysate.

Our ChIP-qPCR analysis further demonstrated that KO of ZNF304 reduced H3K27me3 and H2AK119Ub histone marks on the HIV promoter (Fig 6A and 6B). Our results were also validated at the protein level, as protein immunoprecipitation and western blot analysis showed that HA-TRIM28 associated with EZH2/PRC2 in HEK293T cells (Fig 6C) [16, 62]. We could not detect association between endogenous ZNF304 and EZH2, presumably due to the poor quality of the antibodies. Finally, we followed, by ChIP-qPCR, the occupancy of Pol II on the HIV promoter in cells in which ZNF304 had been knocked-out. Our results confirmed that upon ZNF304 depletion, Pol II occupancy levels on the viral promoter increased relative to control cells, in keeping with the activation of HIV gene transcription upon ZNF304 KO (Fig 6D).

### ZNF304 promotes HIV latency by modulating the kinetics of re-entry of cells into HIV latency

**Fig 7:**
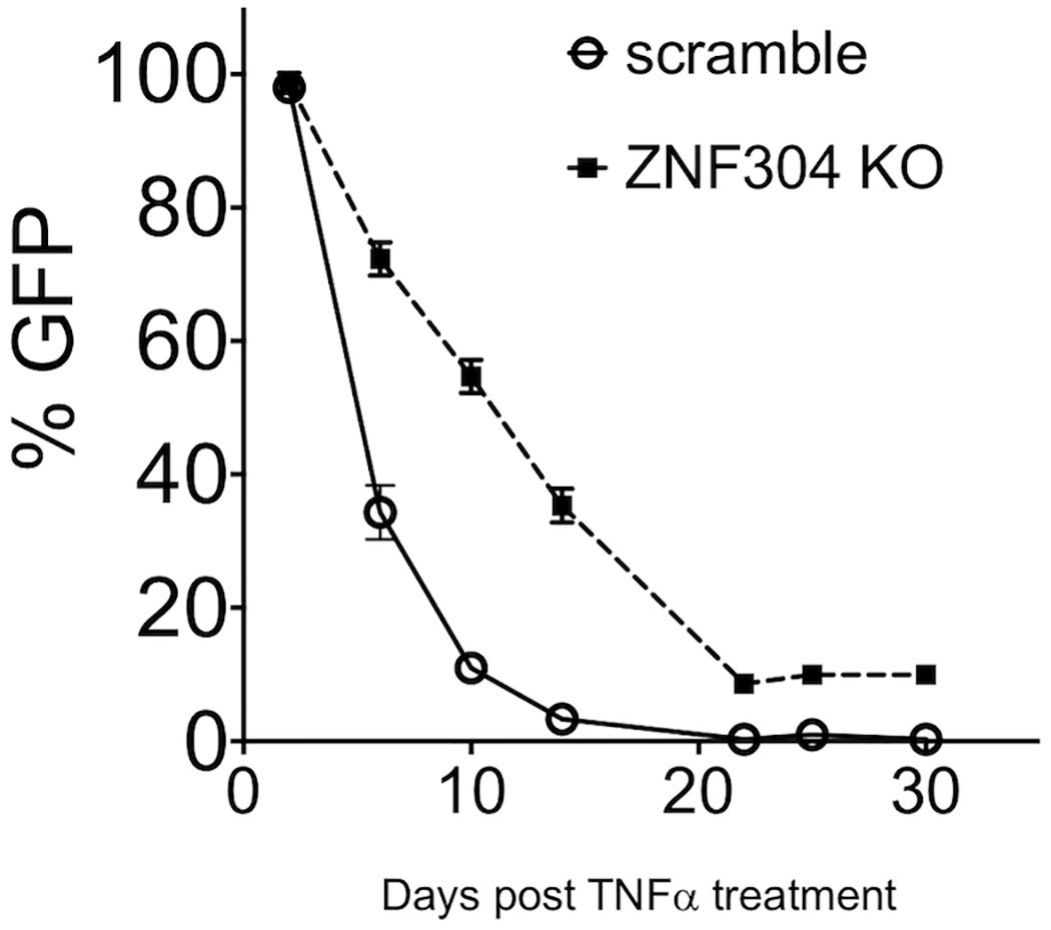
Depletion of ZNF304 expression delays the establishment of HIV latency. Control (circles and solid line) and ZNF304-depleted 2D10 cells (squares and dashed line) were treated with TNFα, and 24 h post cell activation infected cells were sorted based on their GFP expression (day 0). Cells were then grown for the indicated days post infection to allow them to gradually enter viral latency. GFP expression was monitored at the indicated time points by FACS analysis as a reference for entry into viral latency.

To monitor the effects of ZNF304 in enhancing HIV latency, we followed the kinetics of re-entry of activated HIV infected cells into a latency state in control or ZNF304-depleted cells. 2D10 T cells were activated by TNFα, and activated cells were then sorted based on their GFP expression with the aim to isolate cells that harbored a transcriptionally active HIV. Cells were then grown for 30 days, during which their kinetics of re-entry into a latency state was followed by FACS. Our results showed that following TNFα activation, HIV in control cells that expressed scramble sgRNA reached latency within 20 days, while the virus remained active in ZNF304-depleted cells in this time frame. Moreover, in ZNF304 KO cells, HIV remained activated 30 days post TNFα cell activation, as 10% of cells still expressed the viral GFP reporter gene (Fig 7).

### ZNF304 silences HIV transcription in primary CD4^+^ T cells

**Fig 8:**
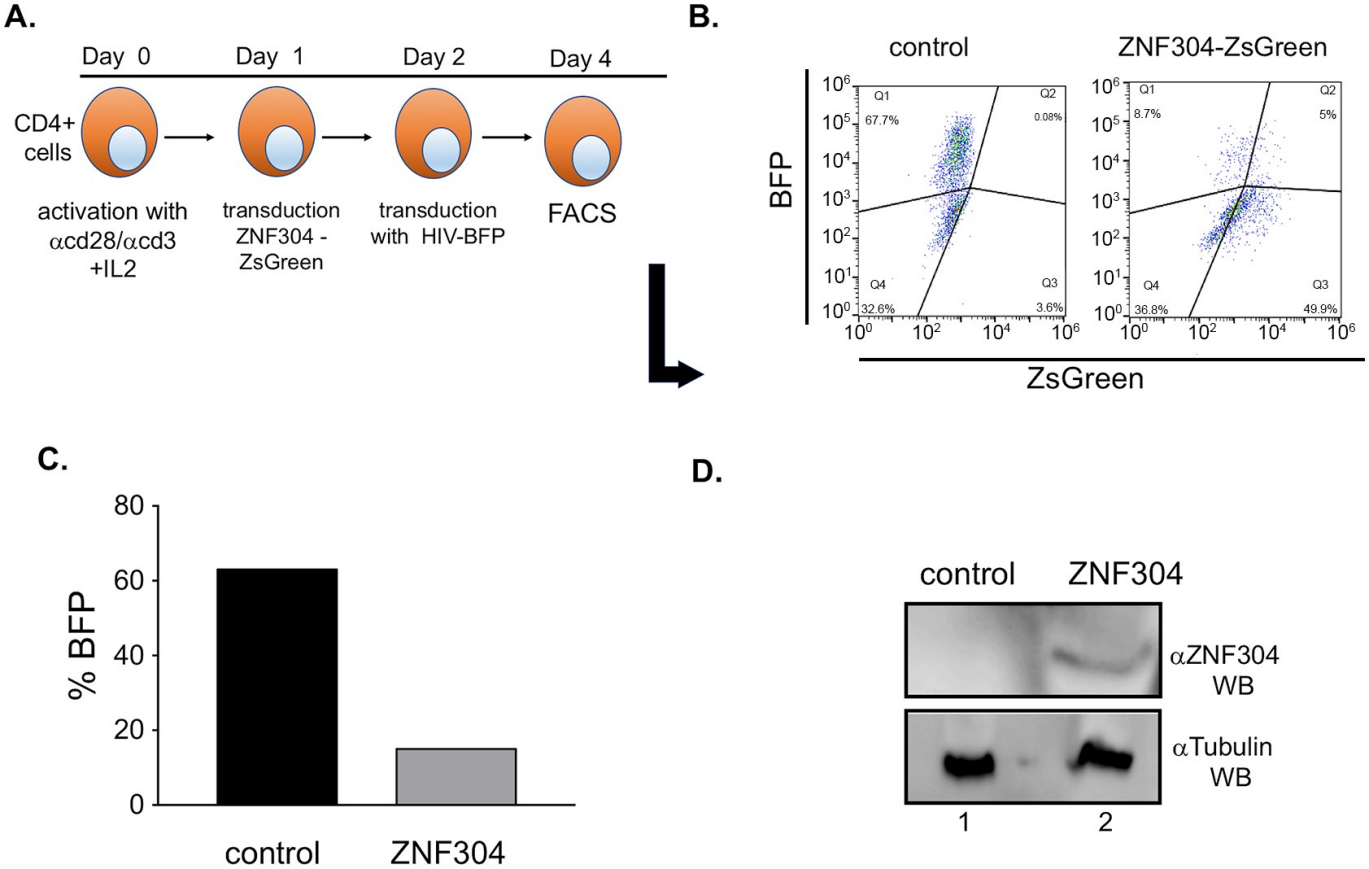
ZNF304 silences HIV transcription in primary CD4^+^ T cells. **A**. Experimental scheme—Naïve CD4^+^ cells were isolated from naïve PBMCs using the RosetteSep Human CD4+ T cell enrichment cocktail and expanded with Dynabeads Human T-Activator CD3/CD28 (Gibco) for 24 h. Activated cells were then transduced with equal amounts of a lentivirus, MOI of 1, which drives the expression of ZNF304. 24h later, cells were transduced again with pNL4.3-HIV-BFP. Cells were harvested 48 h after the second transduction and subjected to FACS analysis. **B**. FACS dot blot of activated HIV-infected CD4^+^ primary cells that overexpress ZNF304. **C**. Quantification of the FACS dot blot presenting the silencing of HIV in primary cells, upon expressing ZNF304. **D**. Western blot analysis confirming the expression of ZNF304 (lane 2) using specific ZNF304 IgG. Lane 1—control cells.

Finally, we aimed to verify the role of ZNF304 in silencing HIV gene transcription in primary CD4^+^ cells. To this end, ZNF304 was overexpressed in CD4^+^ T cells that had been purified from naive peripheral blood mononuclear cells (PBMCs) using the RosetteSep human CD4+ T Cell Enrichment Cocktail, followed by activation and expansion of cells with DynabeadsHuman T Activator CD3/CD28. Activated cells were then transduced with a lentivirus that promotes the expression of ZNF304 and of ZsGreen, and 24 h later the cells were further transduced with HIV-BFP lentivirus. Cells were harvested 48 h post transduction and subjected to FACS and western blotting. Our results confirmed that overexpression of ZNF304 silenced HIV gene expression, as could be seen from the lower levels of HIV BFP (Fig 8A and 8B).

## Discussion

Pivotal work on the mechanisms that control HIV gene transcription elongation has paved the way for the understating how metazoan global gene transcription is executed [63–65]. In this context, the role of the viral protein Tat in recruiting SEC and P-TEFb and in promoting Pol II pause-release and elongation of transcription has been well documented [63, 64, 66–69]. As transcription control is key to silencing of HIV gene expression and establishing viral latency, identifying novel host factors that modulate viral latency is of the highest importance.

In this work, we employed whole genome CRISR KO screen for identifying novel host proteins that regulate HIV gene transcription and viral latency. This study complements other CRISPR screens that were previously employed. Each of these screens has contributed to drawing a more coherent picture of the cell protein network that promotes HIV latency. Among the important and most recent screens, Park *et al*. conducted a CRISPR-based genetic screen in an HIV-susceptible T-cell line and primary CD4^+^ cells by using a high-complexity, genome-wide sgRNA library. Their screen identified host genes that confer robust protection from HIV infection when repressed. These included genes encoding the canonical HIV co-receptors, as well as TPST2 and SLC35B2, which are involved in the CCR5 tyrosine sulfonation that facilitates CCR5 recognition by the HIV envelope, and *ALCAM*, which mediates the cell aggregation required for cell-to-cell viral transmission. Importantly, loss of these factors did not impair cell viability, suggesting that these genes could be suitable targets for therapeutic intervention [55]. Haung *et al*. also performed a CRISPR KO screen and identified the MINA53 histone demethylase, which promotes HIV latency by demethylating H3K36me3 [52]. Similarly, Rathore *et al*. identified several factors involved in RNA degradation and ubiquitin-mediated proteolysis as HIV-1 latency regulators [54]. Finally, by employing a CRISPRi screen, Lu *et al*. identified a key role for the proteasome in maintaining viral latency. Downregulating the proteasome elevated the levels of ELL2 and ELL2-SECs to enable Tat-transactivation, pointing to the proteasome-ELL2 axis as a key regulator of HIV-1 latency and as a promising target for therapeutic intervention [53].

**Fig 9:**
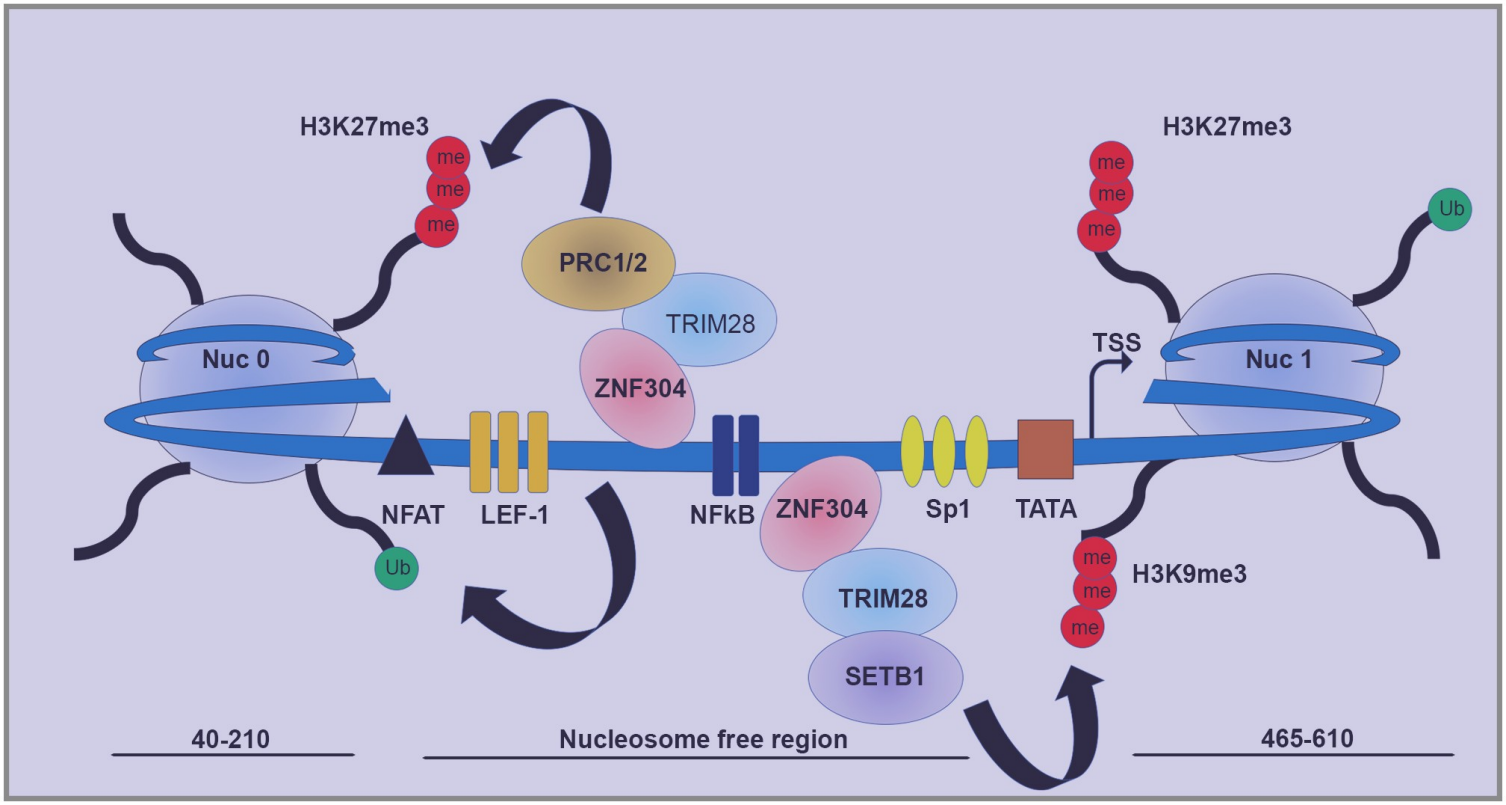
Model for the functional significance of ZNF304 for HIV latency. ZNF304 is tethered to the HIV promoter around NFκB sites, while associating with TRIM28 and recruiting SETB1 and the PRC1/2 repressive complexes, which deposits the repressive histone marks H3K27me3, H3K9me3, and H2AK119ub to silence HIV gene expression.

To the above list, our comprehensive CRISPR KO screen adds novel host factors with a potential role in HIV gene transcription regulation and viral latency. Gene hits, ARL-16, ATF1, CGREF1, and USMG5, have been shown to repress HIV gene transcription and, potentially, viral latency, but their mechanistic mode of action is still under investigation. In addition to the above four hits, ZNF304 was our top hit, as it was shown to silence HIV gene transcription and to occupy the HIV promoter (Figs 3 and 4). In monitoring the expression of ZNF304 and its promoter occupancy during active and latent states of HIV infection, we found that following activation of HIV by TNFα, ZNF304 expression was enhanced. However, while HIV gene transcription took place within 24 h after cell activation, ZNF304 expression peaked later, and reached its peak on day 10 post TNFα treatment, imposing its silencing effects from that day onwards (Fig 3). No enhancement of ZNF304 mRNA levels was observed in non-infected Jurkat cells, implying that ZNF304 expression is regulated by HIV. Further studies are required to define the precise triggers and signaling mechanism by which ZNF304 expression is induced after HIV-1 infection. Mechanistically, we suggest that ZNF304 silences HIV gene transcription through associating with TRIM28 and mediating the recruitment of SETB1, PRC1 and PRC2, thereby promoting histone lysine tri-methylation at H3K9 or at H3K27, respectively (Fig 9). ZNF304 thus acts similarly to other previously identified ZNFs that recruit SETB1 to retroviral promoters via TRIM28/KAP1 [40, 45]. Depletion of TRIM28 or SETB1 in mouse embryonic stem cells, for example, led to a loss of H3K9me3 and upregulation of gene expression of both MLV and endogenous retroelements [36–38, 60]. As our findings show that depletion of ZNF304 reduces levels of TRIM28 and SETB1, we report that ZNF304 associates TRIM28 in cells and recruits it to the HIV genome to silence viral gene transcription through SETB1 and H3Ke3 deposition (Figs 4 and 5). We also showed that upon ZNF304 depletion, levels of H3K27me3 were reduced, implying that ZNF304n is involved in PRC2 recruitment to the HIV promoter (Fig 6). KO of ZNF304 also reduced H2AK119ub histone marks of PRC1, respectively, resulting in transcription repression (Fig 6). Moreover, relative to H3K27me3, the depletion of ZNF304 had only minor effects on the enrichment of H3K9me3 levels (Fig 5). These results agree with work of the Karn lab and imply that PRC2 is the primary player in establishing HIV gene silencing and latency relative to the components of the H3K9 machinery [16, 62]. Moreover, our work also demonstrates that of PRC1 and H2AK119Ub are involved in establishing HIV gene silencing. However, unlike Karn’s work that presented a role for EHMT2 in establishing HIV latency, in our model, ZNF304-mediated HIV gene silencing through the deposition of H3K9me1/2 played only a minor role. Depletion of ZNF304 had no effect on the recruitment of euchromatic histone-lysine *N*-methyltransferase 2 (EHMT2; also known as G9a), which promotes H3K9me1 and H3K9me2. Similarly, no change in enrichment levels were detected in the epigenetic regulator HP1, which interacts with G9a. We thus assume that although reported to participate in establishing HIV latency, both G9a (and probably GLP) and HP1 are not part of the ZNF304 protein network (S6 Fig). In addition, while Karn's work showed that JARID2 anchors PRC2, we demonstrate that ZNF304 associates with EZH2 of PRC2 and potentially recruits the complex to the viral promoter [16].

We aimed to confirm our KO and ChIP experiments by demonstrating protein-protein interactions. We showed protein interactions between Flag-ZNF304 and HA-TRIM28, as well as protein interactions between Flag-ZNF304 and endogenous TRIM28 (Fig 5D and 5E). We could not visualize protein interactions between endogenous ZNF304 and TRIM28 proteins, presumably due to poor quality of the ZNF304 IgG. Our data also showed that ectopically expressed HA-TRIM28 associated with endogenous EZH2 of the PRC2 complex (Fig 6C). The fact that in our hands TRIM28 associated with both EZH2 and ZNF304 implies a functional significance for the ZNF304-TRIM28-PRC protein network. Future experiments will be designed to precisely map the proteins that interact with ZNF304 in cells and their functional relevance. Accordingly, our model suggests that ZNF304 is tethered to the HIV promoter via its zinc-finger domain. Deletion of this region in ZNF304 disrupted the silencing effects of ZNF304 on HIV gene transcription. We are currently trying to narrow down the region where ZNF304 binds to the HIV promoter. ZNF304 then recruits TRIM28 through its KRAB motif, which further associates with SETB1 and the PRC complex and promote H3K9me3 and H3K27me3 respectively (Fig 9).

The eradication of the latent HIV reservoir remains a major obstacle towards a complete cure for infection. To successfully eliminate the latent reservoir, it is first necessary to fully understand the mechanisms that promote the establishment and stability of this cell pool and to identify the host factors that modulate viral latency. These host factors can serve as potential targets for modulating the latent HIV reservoir and ultimately for eradicating it. Herein, we showed that ZNF304 silences HIV gene expression in T-cell models and primary cells. On the basis of our results, new drugs could be developed for administration per se or in combination with already established regimens to optimally and specifically activate HIV gene expression. We conclude that in our search for optimal protocols that would reactivate the virus from its latent state but would cause only minimal global cell activation, targeting ZNF304 expression could be considered as a potential therapeutic strategy that could be integrated into current regimens for battling HIV latency [1, 70].

## Materials and methods

### Cells

The Jurkat T-lymphocyte cell line 2D10 was maintained in RMPI medium (Gibco), supplemented with 10% fetal bovine serum, 2 mg/ml l-glutamine, penicillin-streptomycin, and non-essential amino acids (Sigma, M7145). Cells were cultured at 37 °C with 5% CO_2_. 2D10 cells express HIV-LTR-2dGFP and represent a latent cell model (S1 Fig) [57]. The human embryonic kidney HEK293T (ATCC; CRL11268) cell line that was used for protein interaction analysis and for luciferase assays was maintained in DMEM complete medium (Gibco). Jurkat T cells were transduced with HIV LTR-Luc or HIV LTR Luc Tat.

### CRISPR screen using the GeCKO library

We utilized a CRISPR-based approach to identify novel host proteins that are involved in regulating HIV gene expression and viral latency. To this end, we generated Jurkat cells that express Cas9-Flag (S6 Fig, showing western blot for Cas9). Stable cells were then transduced with a lentivirus expressing HIV-Tat-BFP and sorted on the basis of their reporter expression at 72 h post transduction to obtain a cell population that expressed BFP. Thereafter, 1.3×10^8^ cells were transduced with a lentivirus that expressed the GeCKO whole genome sgRNA library (number of cells corresponded to ×300 the size of the GeCKO library at MOI of 0.3 infection). GeCKO (a generous gift from the laboratory of Ophir Shalem) consists of multiple sgRNA guides that target a total of 19,052 human genes. For each gene, there are six sgRNA constructs, along with 1864 sgRNAs against miRNA with 4 constructs each, making a total library of 122,417 sgRNA members. Cell transduction of GeCKO lentivirus was performed at an MOI of 0.3, so as to statistically ensure the integration of a single sgRNA per cell and to achieve ×300 library coverage (library size 1.3×10^5^). Following transduction, cells were subjected to puromycin selection to obtain stable cells that expressed both Cas9 and the sgRNA GeCKO library. All cells expressed the BFP reporter gene and were thus transduced with HIV. As a control, cells were also transduced with a lentivirus that expressed scramble sgRNA. GeCKO-expressing cells or control cells were then grown over a period of 30–40 days, allowing them to enter latency, as detected by the loss of BFP expression. Thereafter, 40×10^6^ GeCKO-expressed cells (×300 of library size of 1.3×10^5^ members) or control cells were analyzed by FACS for BFP expression. In the GeCKO expressed cells, 44% were BFP(+), while 45% were BFP(–). In the control cells that expressed scramble sgRNA, 22% were BFP(+) and 76% were BFP(–). From each group of cells, 12×10^6^ (×100 above library size) cells were sent for NGS analysis. sgRNA enrichment was analyzed by the CRISPR analyzer and compared for each group of cells vs the control cells that expressed the entire GeCKO library.

### Production of VSV-G pseudotyped lentiviruses

Single round viral particles were produced in human embryo kidney HEK 293T cells by calcium phosphate-mediated co-transfection of the following DNA plasmids: a transgene lentivector expressing the HIV-LTR-luciferase (or LTR-BFP), pGag-Pol, pRev, pTat and pVSV-G, which codes for the VSV glycoprotein. Viral particles were harvested from culture supernatants 48 h post transfection, spun at 2000 rpm for 10 min to remove cell debris, and filtered through a 0.45-μm filter (Amicon). Lentiviral particles were concentrated by ultra-centrifugation for 2.5 h at 25,000 rpm (Beckman OptimaL 90K ultracentrifuge, SW-28 rotor), and the pellet was re-suspended in PBS. The titer of lentiviruses encoding for the Luc reporter was determined by transduction of Jurkat cells with serial dilutions of the virus stock, followed by Luc reporter assays.

To stably express HA-ZNF304 or its delta ZNF mutant, Jurkat cells were transduced with a lentivirus that drives the expression of the protein under the EF1α promoter. The lentivirus expressing HA-ZNF304 also expressed ZsGreen or puromycin (via IRES), allowing the isolation of stable cells by FACS, either based on their ZsGreen expression or after drug selection.

### Lentiviral transduction and luciferase reporter assays

Cells were transduced with a VSV-G pseudotyped lentivirus expressing the HIV-LTR luciferase transgene. To generate transduced cells that express Tat, cells were also transduced with a lentivirus that expresses HA-Tat-BFP. Cells were sorted on the basis of their BFP expression to isolate Tat-expressing cells. Luciferase readings were normalized to protein expression or to *Renila* as an internal control. Lentiviruses that express HA-ZNF304 or Cas9/sgRNA were used for transduction. Expression of HA-ZNF304 or ZNF304 delta ZNF mutant was driven via the EF1α promoter, and the lentivector also harbored a GFP or puromycin gene that was expressed via IRES. Cells were harvested 48 h post transduction, and their luciferase activity was measured according to the manufacturer’s manual (Promega).

### CRISPR-mediated gene silencing

Cells were transduced with a lentivirus encoding Cas9 and each of two different sgRNAs that target ZNF304 (Addgene #49535). As a control, cells were transduced with a Cas9 encoding virus and a scramble sgRNA. Following lentiviral transduction, cells were cultured in a medium supplemented with 2 μg/ml of puromycin to eliminate non-transduced cells. Single puromycin-resistant clones were obtained by serial dilution in a 96-well plate and then further expanded. Clones were genotyped to confirm gene editing, and depletion of ZNF304 expression was further confirmed by western blotting analysis with a specific anti-ZNF304 antibody (ab#108151). Two different clones where ZNF304 expression was knocked-out were functionally analyzed. Western blot analysis with anti-tubulin IgG confirmed equal protein loading.

### Immunoprecipitation in cells

For monitoring the association between ZNF304 and TRIM28, or between TRIM28 and PRC2, HEK293T cells were transfected with a DNA plasmid expressing HA-TRIM28, and Flag-ZNF304. Cells were harvested and lysed with 500 μl of lysis buffer [0.15% Triton X-100; 20 mM Tris-HCl pH 7.6; 200 mM NaCl; 0.72 mM EDTA; 10% glycerol; 1 mM DTT, supplemented with protease inhibitors cocktail (Sigma; 1:200 dilution)]. Lysates were pre-cleared with Protein A-Sepharose beads (Invitrogen) and then incubated on ice for 1 h, followed by centrifugation at 14,000 rpm for 10 min at 4°C; 10% of the reaction was used for input analysis. Cleared supernatants were incubated with 1 μg of anti-Flag IgG (M2-Sigma; A2220) overnight with gentle rocking. The next day, the IP sample was analyzed by SDS-PAGE, followed by western blot using anti-HA IgG (Abcam #9110). Similar experimental design was employed to detect association between Flag-ZNF304 and endogenous TRIM28, using Flag IgG (M2-Sigma; A2220) for IP and TRIM28 IgG (ab22553) for western blot. For investigating the association between HA-TRIM28 and EZH2, the cell lysate was immunoprecipitated with HA-IgG, and western blotting was performed with EZH2 IgG (Millipore 07689).

### Cell-based latency re-entry assays

Control 2D10 cells or cells in which ZNF304 expression had been depleted were activated with TNFα and then sorted on the basis of GFP expression to obtain cells that express GFP. Cells were then cultured for the indicated time post cell activation (Fig 7) to gradually allow them to enter latency, as measured by decrease in GFP expression.

### ChIP-qPCR analysis

Control and ZNF304-depleted 2D10 cells were cross-linked with 1% formaldehyde for 10 min, and after washing with PBS, cross-linking was stopped by adding glycine (0.125 M; 5 min). Cells were then lysed for 10 min on ice in 500 μl of lysis buffer (50 mM HEPES pH-7.5, 140 mM NaCl, 1% Triton X-100, 1mM EDTA, 0,1% SDS and 1% protease inhibitor cocktail), and the nuclear pellets were collected. DNA was fragmented by sonication at the following settings: amplitude 40%, for 10 cycles 20 sec on/40 sec off)Sonics Vibra Cell(. Samples were centrifuged (15 min, 14,000 rpm, 4 °C), and the soluble chromatin fraction (50 μg) was collected and immunoprecipitated overnight with 5 μg of one of the following antibodies: ZNF304 IgG (ab108151), anti H3K27me3 IgG (ab6002); anti H3K9me3 IgG (ab8898); anti CTD IgG (ab817); anti G9a IgG (ab40542); anti HP1 IgG (ab77256); anti ESET (Millipore 071568); or SETB1 IgG (ab12317). Precipitated DNA fragments were then extracted with phenol-chloroform and quantified by qPCR with the primers specifically located on the NFκB region at the LTR promoter (see S2 Fig for primer positions):

**NFκB** forward: 5’ CTGACATCGAGCTTGCTACA -3’
**NFκB** Reverse: 5’—CAGGCTCAGATCTGGTCTAAC -3’
**Rev** forward:—5’ TGATTGTAACGAGGATTGTG-3’
**Rev** Reverse: 5’–TTCTTTAGTTCCTGACTCCA-3’
**Env** forward– 5’-TGAGGGACAATTGGAGAAGTGA-3’
**Env** Reverse: 5’-TCTGCACCACTCTTCTCTTTGC-3’
**GAPDH** Forward—5’ AGCCACATCGCTCAGACAC -3’
**GAPDH** Reverse—5’GCCCAAACGACCAAATCC -3’

All signals were normalized relative to the input DNA. ChIP assays were also performed with normal rabbit or mouse IgG as negative controls.

### Effects of ZNF304 in CD4^+^ primary cells

Naïve CD4^+^ T cells were isolated from peripheral blood mononuclear cells (PMBCs) using the Human CD4+ T cell Enrichment Cocktail (StemCell Technologies)). Cells were then cultured in complete RPMI supplemented with 20 units/ml of IL-2 and expanded with Dynabeads Human T-Activator CD3/CD28 (Gibco) for 24 h. Next, activated cells were stained with trypan-blue, counted, centrifuged for 5 min at 1500 rpm and room temperature, and finally resuspended in fresh RPMI supplemented with 20 units/ml of IL-2 to a final concentration of 0.5×10^6^ cell/ml. Cells were transduced with a lentivirus that drives the expression of ZNF304 and of ZsGreen. On the following day, cells were further transduced with a pNL4.3-BFP lentivirus at MOI of 1. Forty-eight hours after the second transduction, the cells were analyzed by FACS and western blotting.

## Supporting information

S1 Fig Stable expression of Cas9 in 2D10 cells. 2D10 cells were transduced with pHKO14-Cas9-Flag and were then subjected to blastocydin (10 μg/ml) selection for 14 days until all control cells died. Cas9 stable expression was monitored by western blot using anti-Flag IgG (mouse). **Lane 1**. 2D10 naïve cells. **Lane 2**. 2D10 cells expressing Flag-Cas9.
(TIF)

S2 Fig ZNF304 mRNA levels and protein occupancy following TNFα cell activation. **A**. Schematic representation of the HIV cassette in 2D10 cells and indicated primer pairs A-C that were used for ChIP-qPCR to determine ZNF304 occupancy. **B. ZNF304 occupancy on the HIV promoter**—2D10 cells were treated with TNFα to activate HIV proviral expression (active state). Cells were then let to enter latency state in a period of 3 weeks. GFP (-) cells that did not express HIV-GFP were sorted out and represent—latent state. The two groups of cells were then subjected to ChIP-qPCR on either the HIV LTR promoter or on a GAPDH promoter to examine the occupancy of ZNF304 on the HIV promoter using ZNF304 IgG. Control IgG was used as well for non-specific IP.
(TIF)

S3 Fig Predicted binding sites of ZNF304 in the HIV promoter. Predicted binding sites of ZNF304 in the HIV LTR promoter based on SVM scores using an online tool, which is available at http://compbio.cs.princeton.edu/zf/.
(TIF)

S4 Fig Characterization of Jurkat cells in which ZNF304 expression is knockedout. Genotyping of genomic DNA isolated from two Jurkat-ZNF304 KO clones, where the gene encoding for ZNF304 was disrupted by CRISPR/Cas9. Presented are the nucleotide and amino acid residues of ZNF304 surrounding the region where the sgRNA oligos that targeted ZNF304 were located.
(TIF)

S5 Fig Ectopic overexpression of ZNF304 in HEK293T cells silences HIV gene transcription. **A**. HEK293T cells that stably overexpress HA-ZNF304 cells and control cells were seeded in 24 wells, and transduced on the following day with HIV-Luciferase lentivirus, with or without an additional LTR-Tat-BFP lentivirus. Forty-eight hours post transduction, cells were harvested, and luciferase was read according to the manufacturer's instructions. Luciferase readings were normalized to protein levels and are presented relative to control cells set to 1. Bar graphs show mean values ± SD of three independent experiments. Asterisks indicate different levels of statistical significance as calculated by a two-tailed Student’s t test (** p≤0.01). **B**. Western blot analysis of HA-ZNF304 with HA IgG. **C. Overexpression of ZNF304 in TZM cells silences HIV gene transcription**. TZM cells were seeded in 24 wells, and the following day cells were transduced with increasing amounts of a lentivirus that overexpresses HA-ZNF304 (indicated in μl). Eight hours post transduction, cells were transduced with or without a lentivirus that expresses HIV LTR-Tat. Forty-eight hours later, cells were harvest for the Luciferase assay. Data are presented as readings normalized to protein levels and shown as relative readings with no ZNF304 set to 100. **D**. **ZNF304 mutant that is deleted of its ZNF motif does not silence HIV gene transcription**–Jurkat cells stably expressing a ZNF304 mutant that is deleted from its ZNF motif were further transduced with HIV-Luc. For expression of Tat, cells were transduced with lentivirus expressing HIV Tat. Forty-eight hours later, cells were harvested for luciferase assay. Data are presented as readings normalized to protein levels and shown as relative Tat transactivation, where control cells were set to 1. Bar graphs show mean values ± SD of three independent experiments.
(TIF)

S6 Fig Depletion of ZNF304 does not affect recruitment of G9a and HP1 to the HIV promoter. ChIP material was isolated from control or ZNF304-depleted T cells. Immunoprecipitation was conducted with the methyltransferase antibody G9a (**A**) or HP1 (**B**). Non-specific rabbit IgG (black bars) was used as control. qPCR on IP samples was conducted with primers located on the HIV promoter, and signals are presented as a percentage of input. Error bars represent means ± SD of three independent qPCR reactions. Asterisks indicate different levels of statistical significance as calculated by a two-tailed Student's t test (*p≤0.1).
(TIF)
