# Supplementary figures and images for: Genome-wide CRISPR knockout screen identifies ZNF304 as a silencer of HIV transcription that promotes viral latency
Source: PLoS Pathog. 2020 Sep 21;16(9):e1008834. doi: 10.1371/journal.ppat.1008834 (PMC7529202; doi:10.1371/journal.ppat.1008834)

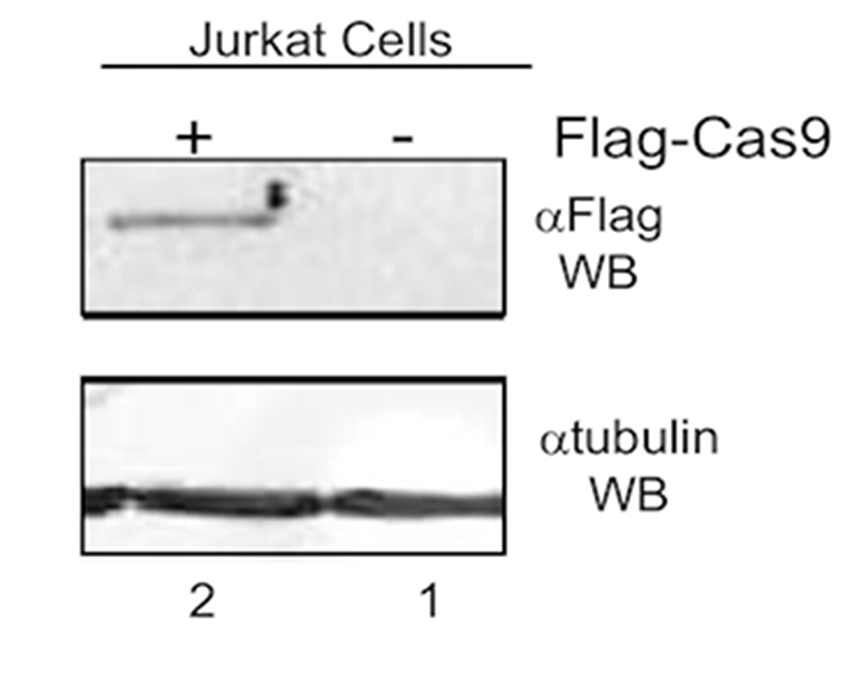

Supplement: S1 Fig — 2D10 cells were transduced with pHKO14-Cas9-Flag and were then subjected to blastocydin (10 μg/ml) selection for 14 days until all control cells died. Cas9 stable expression was monitored by western blot using anti-Flag IgG (mouse). Lane 1. 2D10 naïve cells. Lane 2. 2D10 cells expressing Flag-Cas9. (TIF) [file ppat.1008834.s001.tif]

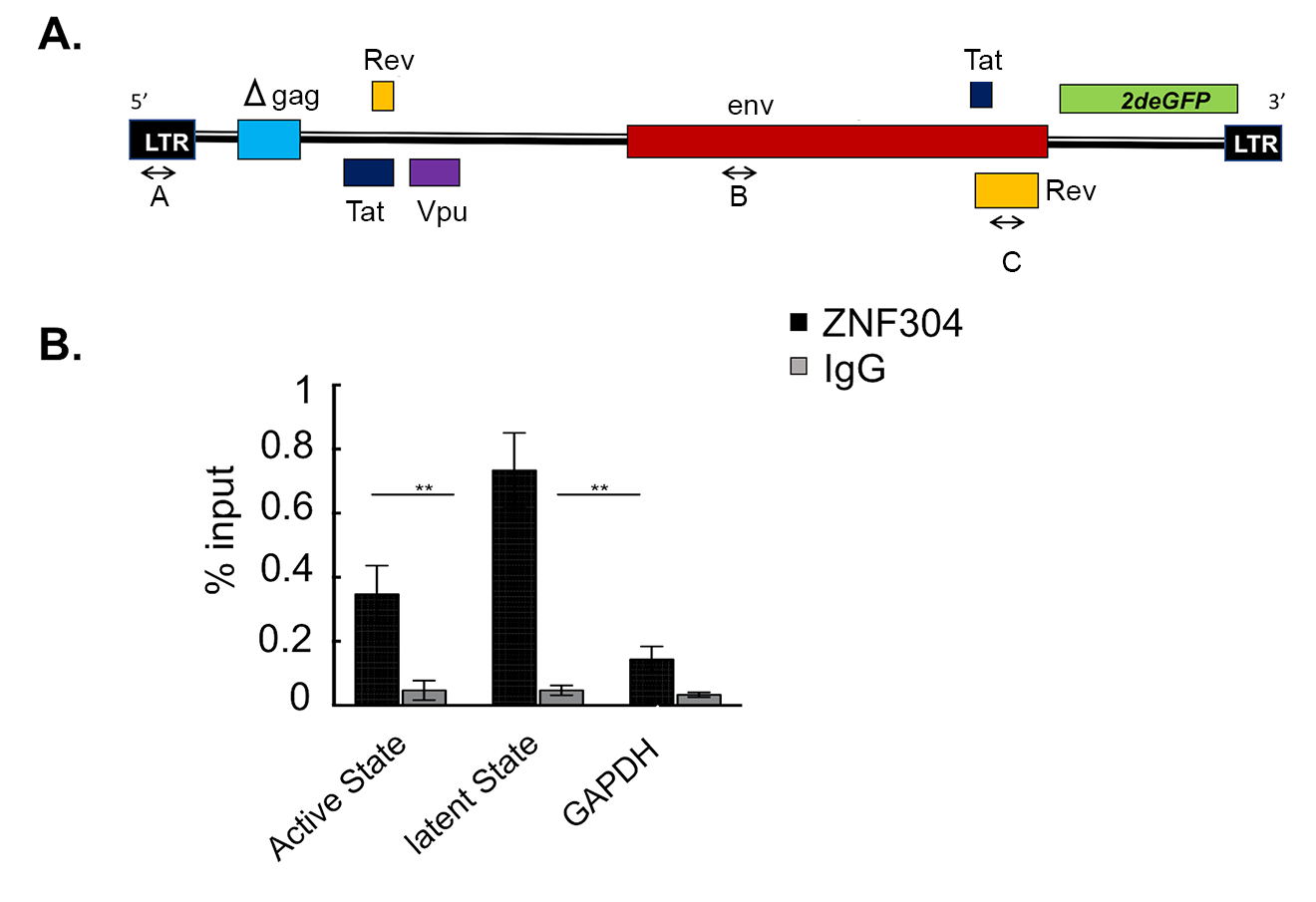

Supplement: S2 Fig — A. Schematic representation of the HIV cassette in 2D10 cells and indicated primer pairs A-C that were used for ChIP-qPCR to determine ZNF304 occupancy. B. ZNF304 occupancy on the HIV promoter—2D10 cells were treated with TNFα to activate HIV proviral expression (active state). Cells were then let to enter latency state in a period of 3 weeks. GFP (-) cells that did not express HIV-GFP were sorted out and represent—latent state. The two groups of cells were then subjected to ChIP-qPCR on either the HIV LTR promoter or on a GAPDH promoter to examine the occupancy of ZNF304 on the HIV promoter using ZNF304 IgG. Control IgG was used as well for non-specific IP. (TIF) [file ppat.1008834.s002.tif]

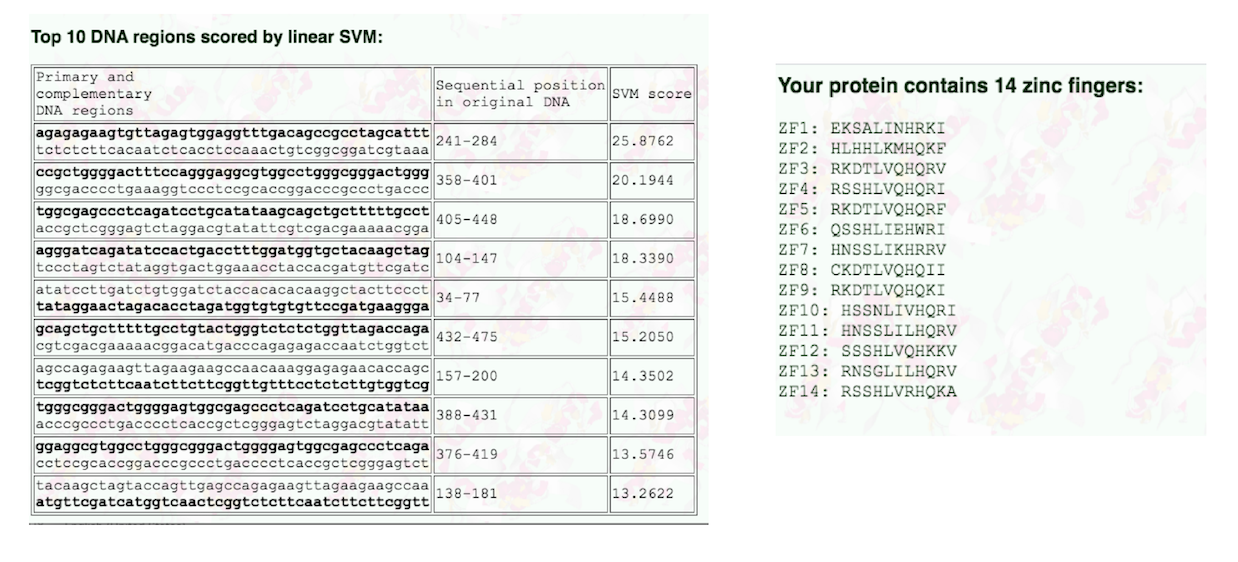

Supplement: S3 Fig — Predicted binding sites of ZNF304 in the HIV LTR promoter based on SVM scores using an online tool, which is available at http://compbio.cs.princeton.edu/zf/. (TIF) [file ppat.1008834.s003.tif]

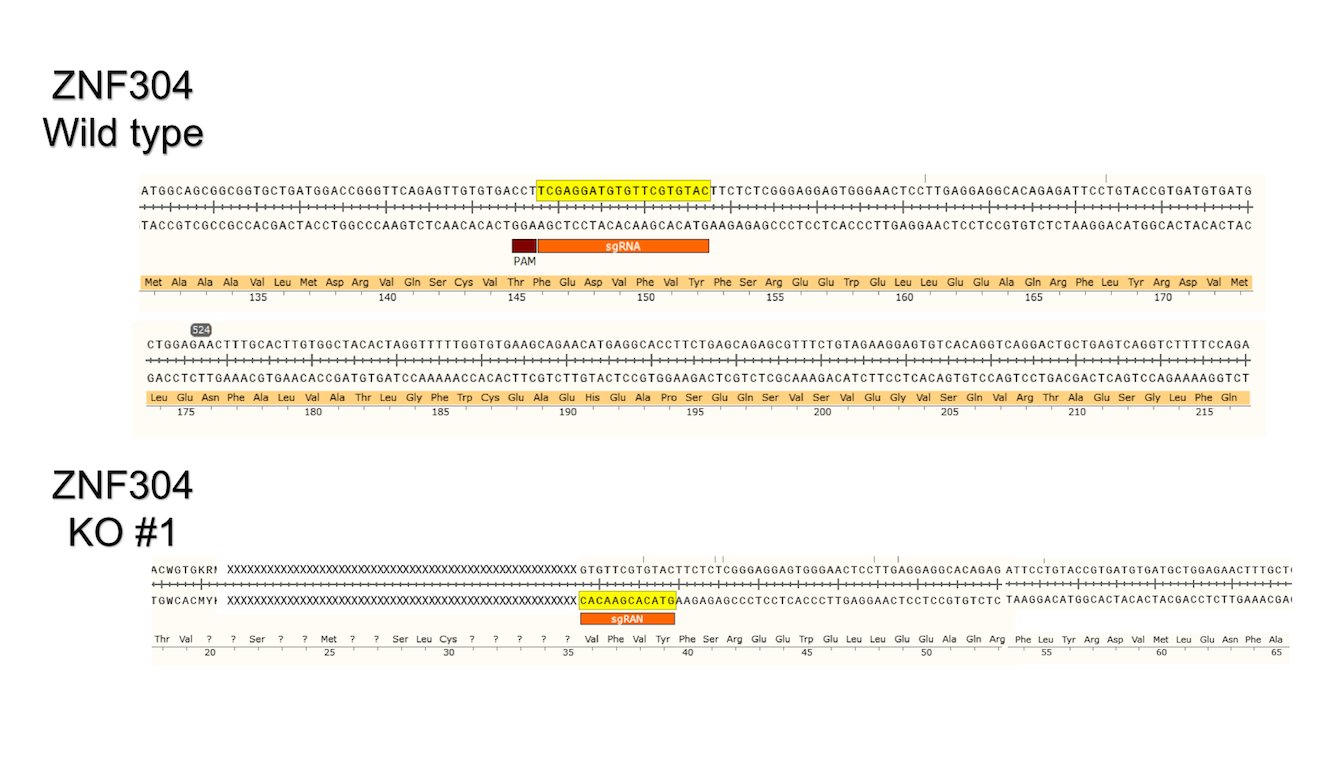

Supplement: S4 Fig — Genotyping of genomic DNA isolated from two Jurkat-ZNF304 KO clones, where the gene encoding for ZNF304 was disrupted by CRISPR/Cas9. Presented are the nucleotide and amino acid residues of ZNF304 surrounding the region where the sgRNA oligos that targeted ZNF304 were located. (TIF) [file ppat.1008834.s004.tif]

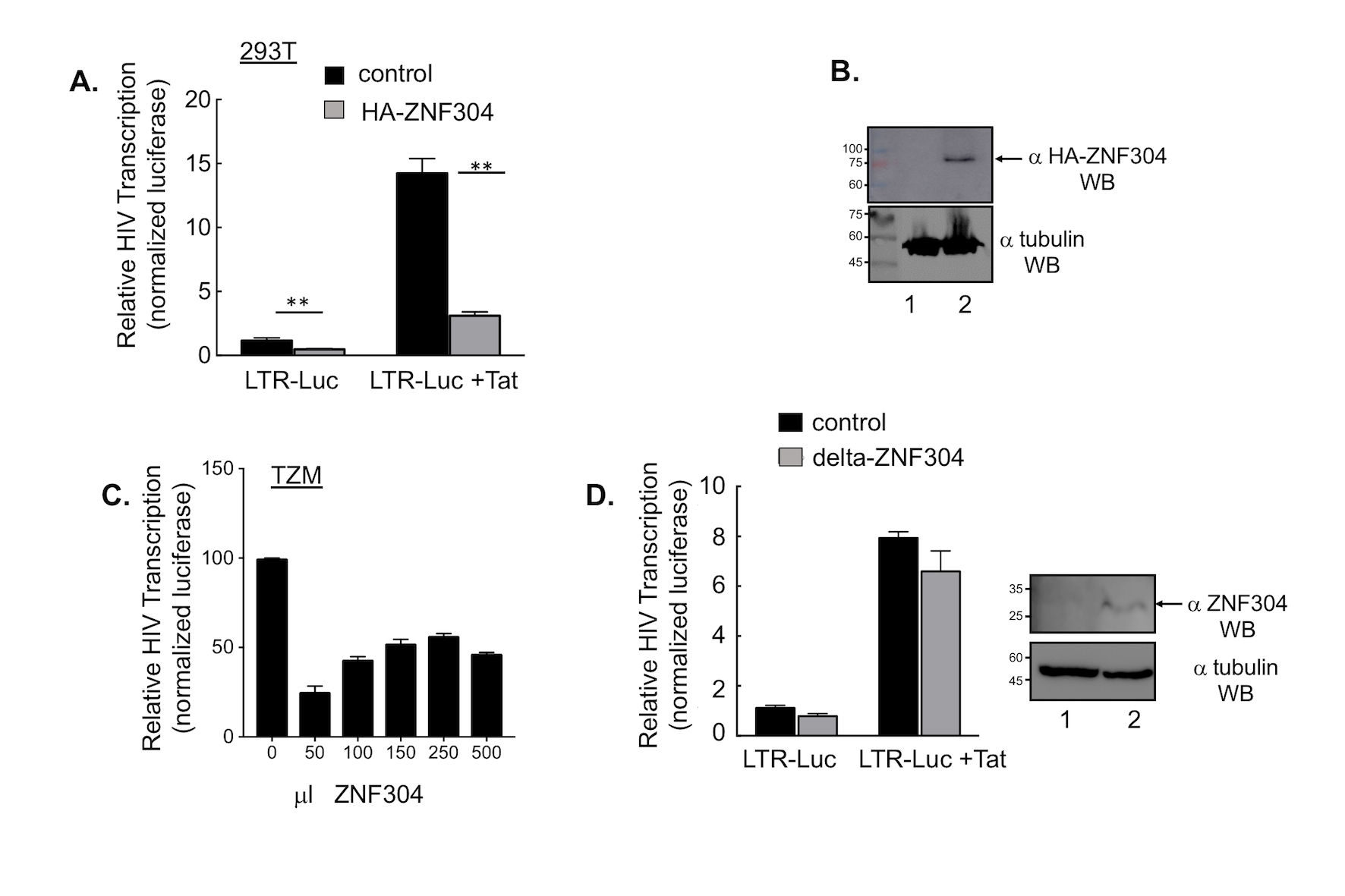

Supplement: S5 Fig — A. HEK293T cells that stably overexpress HA-ZNF304 cells and control cells were seeded in 24 wells, and transduced on the following day with HIV-Luciferase lentivirus, with or without an additional LTR-Tat-BFP lentivirus. Forty-eight hours post transduction, cells were harvested, and luciferase was read according to the manufacturer's instructions. Luciferase readings were normalized to protein levels and are presented relative to control cells set to 1. Bar graphs show mean values ± SD of three independent experiments. Asterisks indicate different levels of statistical significance as calculated by a two-tailed Student’s t test (** p≤0.01). B. Western blot analysis of HA-ZNF304 with HA IgG. C. Overexpression of ZNF304 in TZM cells silences HIV gene transcription. TZM cells were seeded in 24 wells, and the following day cells were transduced with increasing amounts of a lentivirus that overexpresses HA-ZNF304 (indicated in μl). Eight hours post transduction, cells were transduced with or without a lentivirus that expresses HIV LTR-Tat. Forty-eight hours later, cells were harvest for the Luciferase assay. Data are presented as readings normalized to protein levels and shown as relative readings with no ZNF304 set to 100. D. ZNF304 mutant that is deleted of its ZNF motif does not silence HIV gene transcription–Jurkat cells stably expressing a ZNF304 mutant that is deleted from its ZNF motif were further transduced with HIV-Luc. For expression of Tat, cells were transduced with lentivirus expressing HIV Tat. Forty-eight hours later, cells were harvested for luciferase assay. Data are presented as readings normalized to protein levels and shown as relative Tat transactivation, where control cells were set to 1. Bar graphs show mean values ± SD of three independent experiments. (TIF) [file ppat.1008834.s005.tif]

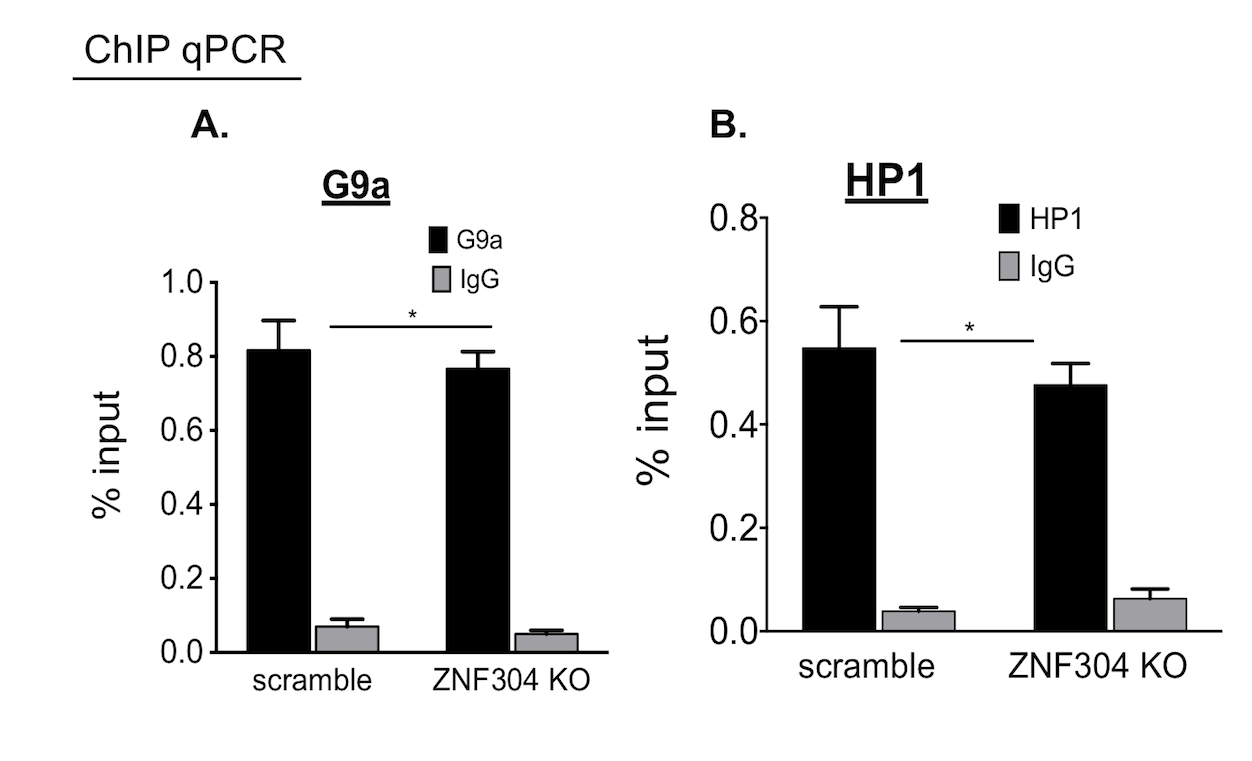

Supplement: S6 Fig — ChIP material was isolated from control or ZNF304-depleted T cells. Immunoprecipitation was conducted with the methyltransferase antibody G9a (A) or HP1 (B). Non-specific rabbit IgG (black bars) was used as control. qPCR on IP samples was conducted with primers located on the HIV promoter, and signals are presented as a percentage of input. Error bars represent means ± SD of three independent qPCR reactions. Asterisks indicate different levels of statistical significance as calculated by a two-tailed Student's t test (*p≤0.1). (TIF) [file ppat.1008834.s006.tif]
